# The Role of Sport Coaches in Promoting the Health and Wellbeing of Athletes with Developmental Disabilities

**DOI:** 10.3390/ijerph23050620

**Published:** 2026-05-08

**Authors:** Roy McConkey, Fiona Murray

**Affiliations:** 1 Institute of Nursing and Health Research, Ulster University, Belfast BT15 1ED, UK; 2 Special Olympics International, Washington, DC 20027, USA; fmurray@specialolympics.org

**Keywords:** intellectual disability, health promotion, public health, sports, coaches, athletes, community-based, family, LMICs

## Abstract

**Highlights:**

**Public health relevance—How does this work relate to a public health issue?**

- People with intellectual disability experience health inequalities world-wide.
- Regular sporting activities in community settings have potential to address their health needs.

**Public health significance—Why is this work of significance to public health?**

- Using qualitative group interviews, this descriptive study obtains the experiences and views of people with intellectual disability and their sports coaches in Special Olympics about their health needs.
- Actions taken by sports coaches to promote the health and wellbeing of their athletes were identified.

**Public health implications—What are the key implications or messages for practitioners, policymakers and/or researchers in public health?**

- Sports coaches should receive training and resources to assist them to merge health plans for individual athletes with their sports training.
- Ways of motivating athletes to take responsibility for developing a healthy lifestyle need to be explored and evaluated.

**Abstract:**

Background: Children and adults with disabilities are widely acknowledged to have poorer health and emotional wellbeing than their non-disabled peers, which is further compounded by less access to health services and health-promoting activities. A relatively untried solution is to mobilize community initiatives such as sports to promote better health. Method: Special Olympics (SO) is an international sports organization present in over 200 countries and jurisdictions, engaging with just under four million athletes with intellectual disabilities annually. Research on the perceptions of sports coaches around incorporating health promotion within their sports training has been scarce. Likewise, little attention has been paid to identifying athletes’ understanding of what health means to them and actions that would make them healthier. A qualitative, descriptive study was conducted with eight national SO programs involving 62 coaches and 47 athletes. Group interviews were conducted via Zoom and a thematic content analysis was made of their responses. Results: In all countries, coaches and athletes agreed that the most common needs were healthy eating, healthy weight and exercise. Good mental wellbeing and sleeping well were also named. Ideas were sought from both sets of participants regarding how coaches could assist their athletes to attain better health and the barriers they might face in doing so. Conclusions: Three main conclusions emerged. Athletes and coaches were aware of health deficits and knew of ways to reduce them. Both appreciated the contribution that coaches could make through motivating athletes and providing training activities but were dependent on suitable resources being available to them. Engagement with families and available health and social care services was essential. Health-oriented, sporting activities offer promise in improving the health and wellbeing of persons with developmental disabilities, particularly in less affluent countries with fewer health professionals and poorly developed primary care services.

## 1. Introduction

The physical and emotional wellbeing of persons with disabilities is widely reported to be poorer than their non-disabled peers, especially in terms of elevated levels of obesity, poor nutrition and lack of physical exercise [1]. For persons with intellectual disabilities, the difference is particularly marked [2]. In part, this arises from inequitable access to health services which happens in more affluent countries [3], but it is more evident in poorer nations with less developed primary care and limited health promotion opportunities [4]. Small-scale efforts to improve the health and wellbeing of persons with intellectual disabilities are commonly reported in the international literature but with mixed success, especially in terms of the sustainability of interventions over longer time periods [5].

To date, most attempts at health promotion for this population have been undertaken by specialist health services with limited engagement with primary healthcare [6]. Furthermore, a public health approach has been rarely tried, other than adaptations of information resources which have had limited impact [7]. A relatively untried solution is to mobilize community initiatives—such as sports—to promote better health, as advocated by the World Health Organization (WHO) [8] who affirmed “the need to ensure and prioritize provision of community and grassroots sport, exercise, and active-recreation opportunities that are inclusive and accessible to all members of the community, particularly in disadvantaged communities and for those who are least active” (p. 73).

Special Olympics (SO) is an international sports organization present in over 190 countries, engaging around five million athletes annually [9]. Various projects aimed at improving athletes’ health have been developed by SO International, such as health screenings at competitions, training opportunities for athletes and Fitness through Sports [10]. They have been mainly parallel activities to the regular training and competitions led by coaches. Nonetheless, when specific health promotion interventions have been undertaken with SO athletes, they reported improved perceived health, reduced body weight, increased fiber intake, improved self-confidence and more positive attitudes toward exercise [11,12].

There are examples in the literature of how mainstream sports clubs have addressed the health needs of their members and the benefits that accrue to their social, mental and physical health. Additional benefits were increased membership and higher motivation for coaches to continue coaching [13]. Indeed, the European Commission in their White Paper on Sport noted the following: “as a tool for health-enhancing physical activity, the sport movement has a greater influence than any other social movement” (p. 3) [14]. This has led others to call for greater recognition for the role sports clubs and, especially, coaches can play in public health [15,16].

Arguably the health gains for people with intellectual disabilities arising from participation in SO could be enhanced if health promotion was adopted by coaches as a key aspect of their role. However, a vital starting point would be to obtain the views and lived experience of both coaches and their athletes which, to date, has been limited [17], in particular, identifying athletes’ understanding of what health means to them and the actions that would make them healthier. Likewise, the perceptions of their sports coaches around incorporating health promotion within their sports training remains scant. Equally so, their perceptions of the facilitators and barriers to implementing a more proactive approach to health promotion should be sought [18].

### Aims of the Study

The study had two main aims. First, to identify the health needs of SO athletes as perceived by athletes and coaches. Second, to discover the role that coaches are playing, or could play in promoting the health and wellbeing of their athletes. This aim would also embrace the barriers likely to be encountered. Again, reports from both athletes and coaches would be sought.

Two further ambitions were incorporated into the design of the study. We wanted to explore the value of using a participatory approach in which sports personnel in each country would be enlisted as co-researchers [19]. Their involvement could prepare regional and national SO programs to undertake future research and evaluation projects. Second, we wanted to recruit a transnational sample of coaches and athletes in order to assess possible variations across more and less affluent nations. A qualitative descriptive study design was adopted, drawing on the lived experience of the chosen participants within a global context [20].

## 2. Materials and Methods

The overall project was supported by a grant to Special Olympics International (SOI) from the Stavros Niarchos Foundation. The first author was commissioned to co-ordinate the development and implementation of the study. Ethical approval was obtained from Ulster University, UK. Easy-read versions of the information sheet describing the study and the consent forms that were obtained from athletes and coaches were prepared. These were prepared in English and translated into local languages as required.

Invitations for SO programs to participate in the study were sent to the Regional SO leads in Africa, Asia–Pacific, Europe and South America, who in turn notified the national SO programs in their region who engage in health promotion activities: around 60 in total. Eight programs responded to our invitation to become partners and agreed to take part. They were Nigeria, Senegal, South Africa, Fiji, New Zealand, Ireland, Great Britain and Mexico. Together these programs had nearly 130,000 athletes enrolled and over 19,000 coaches.

### 2.1. Project Personnel

Each national program nominated a lead person or persons for the study who were paid or volunteer members of staff with responsibilities for sports or health. S/he linked with the project coordinator (the first authors) who co-designed with them the tasks they were requested to undertake and to guide them through. SO regional staff were also available to provide support to their national programs and to offer advice, guidance and reminders on request. Each national program received a grant of US$3000 to cover their costs of staff time and incidentals such as internet and phone calls.

The national program leads personally and purposively recruited up to 10 coaches from a range of sports to participate in the study, including team sports, such as soccer and basketball as well as swimming and athletics. Further criteria were an interest and involvement in promoting the health of their athletes and from a range of backgrounds: sports, health and education. Overall, there was a balance of male and female coaches.

The national leads purposively invited up to six athlete leaders from their country from different sports as per their coach, from varied social backgrounds, mix of genders and with good verbal communication. SO has developed programs for selected athletes covering leadership, personal and professional development training so they are better equipped to hold leadership roles in their Special Olympics Program, community, and workplace. Health matters are included in this training.

All the coaches and athletes who were approached agreed to take part.

### 2.2. Procedures

Group interviews were conducted via Zoom; separately for coaches and for athletes.

The national leads for the project were the group facilitators. In consultation with the project coordinator, the questions, probes and approach to be used on the groups were standardized across the eight country groups. The information that was sought included the health needs of athletes, how they currently promote the health and wellbeing of athletes—what works and what has not worked; the reaction of athletes and carers to health improvement; and the supports coaches would need to further improve their athletes’ health.

A similar process was used with the athlete leaders with broadly the same questions and an emphasis on how athletes could improve their own health and that of other athletes, how coaches can support athletes and how best to motivate athletes to improve their health and well-being.

Participants joined the group call mostly using their Smartphone; however, the connection at times was variable. Meetings generally lasted up to 60 min for coaches and 40 min for athletes (not including introductions). The recordings were transcribed and translated as needed into English using a computer software. The transcripts were then reviewed and corrected by the interviewers.

In total, 62 coaches took part across a wide range of ages, and most have been involved with Special Olympics for over five years. Athletes were recruited in seven countries (except Senegal due to lack of phone connections to the internet) and 47 took part. They included males and females who had been involved with SO for many years (most could not recall when they had started) and participated in a variety of sports as noted above. In total, eight group interviews were conducted with coaches (with 6 to 10 in each) and seven with athletes (with 6 to 7 in each).

### 2.3. Data Analysis

In total, around eight hours of interview data were available for coaches and five hours for athletes. The national leads were first asked to review the transcript of their groups and to make any corrections and clarifications.

The first author then conducted a thematic content analysis using the procedures outlined by Braun and Clarke [21]. The initial codes were identified inductively and informed by the previous literature and the targets for promoting population health and wellbeing [22]. For each main theme, specific examples of subthemes were identified that were further supported by short and longer quotes from individuals in the groups.

The initial analysis was sent to the eight national leads and four regional personnel for checking clarifications and significant omissions, but few changes were made. We assumed that data saturation had been reached given the number of and diversity across both groups of participants, at least within the context of Special Olympics sports.

## 3. Results

### 3.1. Health Needs of Athletes

Table 1 summarizes the main themes describing athlete health needs and commonly mentioned suggestions for how the needs can be addressed, which is presented in the form of short quotes from participants in the groups. In the table, the suggestions that are common to both groups are aligned for athletes and coaches to provide an indication of the extent of agreement. Those that have a blank space next to them were proposed solely by one group of informants.

The most frequently mentioned health issues in the group interviews were healthy weight, healthy eating and drinking, and exercising. The following quotations, expand on the summary given in Table 1.

*You must exercise daily must drink lots of water. each and every day exercising… eat healthy food. You mustn’t eat a lot of food”. (Athlete, Mexico).*

*One (athlete) we have, he was overweight when he joined us and he’s put on 40 kilos since then … he goes to the supermarket whenever he wants. (Coach New Zealand)*

*My athletes know not to bring fuzzy juice, energy drinks or chocolate bars or things like that it’s got to be food that will help them but unfortunately before they come, they go to McDonalds. (Coach, Great Britain)*

*Try as much as possible to take the right food, mostly fruit and vegetable things that will help me boost my immune system. (Athlete, Nigeria).*

*We are so blessed with vegetables and a lot of greens, so with parents we asked them for our athletes to have a healthy food that is homegrown (Coach, Fiji).*

*Go out and do better walking or running and better cycling or anything to keep yourself fit and healthy before you go back into your sports. (Athlete, Ireland).*

*Our boys have been doing a gym program twice a week so they’ll go onto the field and they’ll do push ups and sit ups (Coach, South Africa)*

In addition, athletes and coaches mentioned other health issues that were a concern to them, including mental wellbeing, sleep and access to a doctor or dentist.

*Anxiety sometimes relaxation of mind as well, and so many factors that makes an athlete not to be in fully in good health and by preventing these factors is going to make the athlete to be good, (Athlete, Nigeria)*

*We’re trying to create an environment where they can just come in and relax. Don’t worry about making them stronger, just make sure they’re having a good time, because (it) gets rid of some of that stress and there is definitely a difference (Coach Great Britain).*

*I find it hard to go to bed early and then get up and start doing my work, I find it really hard so. (Athlete, Ireland).*

*Once in a while go for medical check-up. If things detected, to be able to treat that at least… and maybe that is the way they could be supported financially (Athlete, Nigeria).*

*The biggest health problem is 100% dental. In one match, we had to stop because (a player’s) tooth cavity was causing a severe pain (Coach, Mexico)*

*The biggest health and well being concerns for me was always our athletes who live independently and had no access to make wiser choices for themselves, and I’m talking no smoking, and alcohol. (Coach, Great Britain).*

### 3.2. Promoting Health and Wellbeing

The coaches and athletes knew what needed to be done—the challenge was how to do it—and, in particular, how coaches could be of assistance. Listed in Table 2 are the many ideas put forward by coaches and athletes. The ideas that were common from both athletes and coaches are aligned. A blank beside an item came solely from one group.

All coaches felt they had to attend to the health and wellbeing of their athletes as it was essential to their sporting performance, but whenever possible, the athletes should be supported to take responsibility for their own health. Not surprisingly, a major topic of conversation was around how athletes could be motivated to exercise more and to have better nutrition. Linked to this was the need for coaches to enlist the support of parents or other family carers, and in the case of people living independently or in residential accommodation, to partner with paid carers. Ideas for how changes in athletes’ health could be assessed were also noted.

Coaches also spoke about their own need to be better informed and more knowledgeable about how to deal with health issues and suggested ways that this might come about. The importance of using less formal means of promoting healthy lifestyles were also highlighted, such as the example set by coaches, informal conversations and directing them to other agencies to get assistance. The main challenges were: motivating the athletes to change their habits, receiving the support of families, and training coaches in health promotion. Ideas were also shared about how to devise health promotion plans suited to each individual.

Here are examples of the comments made by the participants in the two groups. As the table shows, athletes and coaches often complemented each other with different strategies or with particular ideas.

*I felt alone, abandoned, because I did not feel the support of my father or my coaches. So there must be support, even if they see me like that, because they see me as normal, even if I have a disability, but support is often needed, in addition to sports. (Athlete, Mexico)*

*Our people singularity lacking in initiative. You have to develop that initiative and promote that initiative before they can go and do those peer things. We’re asking them to be self motivated and it’s totally foreign to them (Coach, New Zealand).*

*During the lockdown I not training at all, so the best thing for me was YouTube. I decided no wait anymore I’m gonna start doing push ups, sit ups, you know just motivate myself that’s, the only thing you can do. (Athlete South Africa)*

*If there’s some workouts, awesome exercise that the athletes can do, for example coaches can adapt or show them how to do that exercise. Make it simpler for them, they can do it or change it up completely so its sort of the same exercise but doing a different way, so adapting. (Athlete, New Zealand.*

*Aerobic exercises doesn’t interest them. They get interested if they do a tangible game like if we playing soccer, netball all those things. Once it becomes boring, you do not want to do anything so let’s make exercising fun and exciting (Coach, South Africa)*

*For us, coaches, it will be a boost, if we go through a training. Because during our training sessions with the athletes we will be the first person, they will approach, so it will be of great help if we have some content knowledge of basics. Involving families in the workshops for coaches on health as also gives a boost for parents to broaden the knowledge. Volunteers and donators could also attend… it boosted most of my parents’ athletes to follow the diet and training checklist (Coach, Fiji)*

*If the older athletes will sit down with the younger athletes and say oh look we’ll try this this week for competition and see how we get on and if that works, stay with that fruits and veggies (Athlete Ireland).*

*Ask for some assistance if there is an elder or a partner in their home or neighbor, just to assist my athletes (Coach, South Africa)*

### 3.3. Reservations from Coaches

Some coaches—notably in New Zealand and Great Britain—had reservations as to whether coaches should take on the responsibility of improving the health and wellbeing of their athletes.

*“I don’t think it’s the coaches duty to document the health and well being of athletes, they have enough to be doing in their coaching sessions. I don’t think it’s even appropriate to be keeping records of how they are in their health and well being; you’re there for their sport … but you know the health and well being is I think beyond our remit”.*

Time pressures were also an issue for other coaches.

*For sporting activities it’s only for one to two hours, so now it is very much difficult to check if they are eating properly at home.*

*It really a limit to what we can do as people only see them once a week.*

*How little time coaches specifically have to influence their athletes … how important it is for their carers to support them as families, friends, how big of a role that they actually have to play in education.*

Respect for athletes’ right to privacy and to make their own decision had to also be balanced against coaches’ duty of care for their wellbeing. This is more so with those living independently.

*Some of their parents would consider it quite intrusive if we did try to get involved and help.*

*(The athlete says) I don’t need you to tell me what to do, I can find my priority is independence and my priority is doing the things that I like to do so you’re not telling me that I can’t drink (alcohol).*

*I do have a few live independently and you just couldn’t keep them at it, it is hard work and then it just slipped back very, very easily with them.*

## 4. Discussion

This study was unique in a number of ways. It obtained the experiences and views of athletes with intellectual disabilities allied with similar information from sports coaches. The participants, totaling over 100 persons, were recruited from eight countries world-wide including affluent and less affluent countries. The findings specified the health needs of athletes with intellectual disabilities and also the variety of ways in which sports coaches were promoting, or could promote, the health and wellbeing of athletes with intellectual disabilities. There was little variation across the countries but coaches from less affluent countries were more likely to comment on malnutrition and poverty among their athletes, which is supported by past research [23].

Although no formal evaluation was undertaken with the national leads about their contribution to the study, they spoke about how their personal contacts with many informants led to more open and rich insights. Likewise, they could confirm the validity of the information obtained from their own lived experiences. They felt affirmed through being part of an international study and most were eager to be involved in future initiatives. These echo the experiences of other studies [24].

The study has some limitations that need to be borne in mind for future research. Although Zoom calls had advantages in terms of costs and recruiting participants from dispersed locations within countries, two particular disadvantages were connection troubles, causing people to drop out from the call and having to re-enter, and a lack of interactions among the participants, which limited discussion of contentious issues, notably, whether coaches should get involved in health issues and the right of athletes to make their own decisions regarding their health. It should be borne in mind too that the limited sample from self-selected countries and chosen participants may not be representative of health issues and practices in other SO settings and in other sporting contexts that involve people with intellectual disabilities and coaches. Further research that address these limitations would make a valuable contribution to the literature.

Nevertheless, Table 1 and Table 2 provided a rich source of information that echo many themes identified in population health promotion and the strategies for addressing them. Certainly, three main conclusions can be drawn in relation to Special Olympics, which might also have application for other sports clubs that include people with intellectual disabilities.

Firstly, athletes and coaches were aware of health deficits and knew of ways to reduce them. These aligned with the lifestyle changes promoted for better population health, although some additions were also added, such as personal care and dental care, which may be more pertinent for persons with intellectual disabilities.

Secondly, both groups appreciated the contribution that coaches made or could make to athlete health with the proviso that suitable resources were available to them. They especially valued training opportunities that raise awareness of health issues and increased their knowledge about them, and practical strategies to address them. Nonetheless, the limited contact time they had with athletes meant their influence could be inadequate. Training for coaches is a common theme in past research [25], but it would be important that the content is contextualized within the sporting environments provided by Special Olympics with its combination of recreational and competitive sporting activities and short training sessions [26].

Thirdly, the engagement with families and paid carers as well as available health and social care services was deemed essential by coaches to ensure that any behavioral changes were sustained at home, particularly in relation to healthy eating. Also, that advice was available from them to coaches of specific health conditions. Useful ideas were provided as to how these connections could be made. However, this endorses the need for health promotion within sports to be rooted within a broader socio-ecosystem than the micro-context of the coach and an athlete [26]. Furthermore, a systematic review of factors that influenced the sustainability of community-based, multilevel health promotion activities highlights the importance of broader strategies of ‘Empowerment and Capacity Building’, ‘Participation and Partnerships’, and ‘Community Support’ of sports clubs [27].

An additional challenge remains, namely, motivating athletes to take responsibility for their own health and wellbeing, especially in relation to exercise, healthy eating and healthy weight. A systematic review of studies on self-management by persons with intellectual disabilities identified the usefulness of behavioral change techniques—such as goal setting, incremental steps and charting progress—in relation to an activity of their choice, although none of the studies focused on health issues or were conducted within a sports context [28]. Further health promotion studies used different strategies to motivate participants with intellectual disabilities; including pairing with a non-disabled peer and earning incentives for achieving goals [12]; emphasizing fun and social interaction [17], peer-to-peer support and presence of a care-giver (family or paid supporter) [29] and the development and delivery of an educational module on healthy eating in collaboration with mentors who had intellectual disabilities [30].

It remains uncertain as to how the ideas and intentions identified in this study can be implemented more widely with coaches and athletes within SO clubs. Insights can be gained from the frameworks for implementing public health initiatives such as that developed by Schnell and colleagues [31] and its adaptation to community sporting contexts [26,27]. These stress the importance of adopting a whole systems approach, addressing issues beyond the person-coach dyad in order to build an effective, efficient and sustainable intervention. Most SO clubs, in common with many other community sporting initiatives, rely on voluntary efforts. It is difficult then to prescribe how change agents could emerge in such systems to build greater engagement with health promotion activities. Health promotion initiatives have to be attuned to persons who voluntarily make themselves available. Moreover, SO nationally and regionally is largely a federations of locally autonomous clubs who share a common ethos and sporting practices. Again, any initiative relating to athlete health has to come from themselves, albeit with encouragement and support from the wider SO community and, particularly, other local SO clubs who have embraced health promotion.

Perhaps, focusing first on an athlete’s mental and emotional wellbeing might be more acceptable to coaches as there is stronger evidence for the impact of sports participation—especially team sports—on an athlete’s self-esteem, life satisfaction, reduced levels of depression, anxiety, and stress, and improved social outcomes [32,33]. Research with SO athletes also suggests that they show similar benefits [34]. From these experiences, coaches and athletes could accrue a greater appreciation for the mutual impact between mental and physical wellbeing [35].

Further research and evaluation studies are needed on SO clubs and other community sports clubs who are reliant on volunteer coaches, especially those who have successfully combined sports training with the health promotion of their members. These would provide evidence-based practice that others could emulate in order to realize the potential of sports in promoting better health for persons with intellectual disabilities, in particular, but also for the wider population of children and adults who participate in sports. These would widen our appreciation for how community groups can be mobilized in public health strategies to improve the overall health of their population [36].

## 5. Conclusions

A rich source of information was gathered as to how the health of people with intellectual disabilities could be improved from the perspectives of both athletes and coaches, and the role that sports coaches have played or could play in this. It is likely that all the pertinent themes were identified given the large number of participants recruited from across eight countries globally. The findings confirm that knowledge per se is necessary but insufficient in eliciting lifestyle changes with the athletes. Their motivation to maintain healthy behaviors was an enduring issue. Both athletes and coaches identified the need for training in health issues as well as suitable resources to assist their planning and recording of health goals. Moreover, engagement with families and available health and social care services were essential in supporting the athletes to attain and sustain their chosen goals.

Health-oriented, sporting activities offer promise in improving the health and wellbeing of persons with developmental disabilities, particularly in less affluent countries with fewer health professionals and poorly developed primary care services. However, further research on how local sports club, which are dependent on volunteers, can be supported to introduce and sustain a health focus within their activities is needed. Moreover, a focus on low-resourced communities and marginalized groups would be especially welcome.

## Figures and Tables

**Table 1:** How the health of athletes with intellectual disability could be improved.

Topic	What ATHLETES Said	What COACHES Said
Healthy Weight	Need to lose weight. Go on a diet. See nutritionist to help lose weight. Not enough money to buy food.	Many are overweight and obese, especially female athletes, it affects their performance and their general health. Weight gain during COVID. Malnutrition, dietary deficiencies.
Healthy Eating and Drinks	Too many take-ways, junk food. Too many sweets/candies; cut out sugar. Not eating enough fruit and vegetables. Smaller portions. Stay away from sugary drinks, drinking too much cola. Flavored water can taste better than plain water.	Eating too much junk food Cut out sweets/candies. Use home grown fruit and vegetables. Families give too much food; portions too big. Ban sugary, fizzy and energy drinks Drink plenty of water. Carry a water bottle.
Exercising	Walking, running, cycling Go to the gym. Exercise daily for at least 30 min; build up from 5 min. Try new sports. You can do exercises in your house, in the kitchen. I track my exercise with a fit-bit, step counter. Stretches are good for your muscles.	Fast walking not strolling. Have a gym program with exercises. Dance competitions. Not enough exercise, lack of training outside of Special Olympics. No enthusiasm to do activities, they become lazy.
Mental Wellbeing	Make friends and keep connected. It’s OK to ask for help. Try relaxing music and relaxation activities. Be confident and happy in yourself. Don’t get stressed, be calm and take your time.	Meeting with others. Knowing what to do when you feel down, anxious. Sport and exercise reduce stress; help to relax.
Sleep	Sleep 8 h a day. Stay away from phones and TV before bedtime. I find it hard to go to bed early.	Check if they are getting a good night’s sleep. No cola or energy drinks before bedtime. Involve parents to monitor their sleep.
Personal Hygiene	Practice good hygiene—hand washing, bathing/showering. Check your place is clean.	Skin care -Itching, rashes, boils. Menstrual hygiene. Very long nails. Footwear does not fit.
Access to doctor	Parents do not take them to hospital when ill. Go for check-ups once in a while Take your medication. Provide financial support.	Parents do not follow up when problem identified. Not enough money to buy medicines Cannot pay hospital bills.
Dental Care		Teach how to brush teeth. Check for tooth pain. Bad breath affects close contact sports.
Substance Abuse	Cutting down on the amount of cigarettes, maybe will be good.	Athletes living independently are more likely to smoke and use alcohol.

**Table 2:** How coaches can help athletes to be more healthy.

Action	What ATHLETES Said	What COACHES Said
Motivating Athletes	Make it fun—do happy things like dance, music. Help us to help ourselves. Have support from others. Athlete leaders can support individuals or groups. Keep encouraging one another. Keep in touch through social media—WhatsApp. You Tube videos were great during lockdowns. Keep it simple—no jargon. People have different health issues—be sensitive. Be spiritual—pray for help to perform well. More health campaigns aimed at athletes. Lack of training for athletes and coaches on getting motivated.	Make it fun—don’t scold. Give plenty of praise. Encourage them to take ownership for changing. Use peers as mentors. Organize a small group of training buddies. Identify a support network at home and community. Make use of What’s App and Facebook to keep in contact. Send texts to remind them. Call at their home to check on progress. Provide handouts/videos showing what to do. Focus on one or two goals that are likely to be reached.
Involving families	Talk to our parents to have them help us. Make parents more aware of health needs. Families need to know about our rights and their own rights. Parents need training from experts on healthy options. There are others in the family who could support us—uncles, cousins.	Visit the family home; call them by phone. Ask what problems they see for their child. Be direct—tell them dangers associated with poor habits. Create What’s App Group for families and athletes. Provide families with nutritious food. Invite families to information/ training events. Invite parents to health screening events. Share videos/handouts of activities to do at home.
Training for coaches	Encourage the coaches to get training. Find out about the health issues of their athletes and get training on them. Have more Webinars on health. You Tube has some great videos. Health messengers/athlete leaders can visit clubs to educate everyone about health. Coaches could team up with a health professional.	Make the training practical and interactive. More online courses—provide training manuals. Ensure new coaches get training. Provide refresher training. Organize meetings of coaches to share ideas and experiences. Provide resources—handouts, checklists. Recruit health professionals to assist with health promotion.
Talking with athletes	Don’t give too much information. Show us the techniques, what to do, not just tell us. Offer reassurance that we will be able to change.	Have casual conversations about health in groups and individuals. Find out their health concerns and the help they would like to have. Build a relationship. Be respectful, treat as an adult. Give simple explanations. Be positive. Give praise.
Set an example	Coaches guide us in sports so they can guide us to be healthy. Have a competition within clubs about eating the right food, exercising and so on. Link us up with others who can join us in exercising, going for walks.	A coach should be a role model of healthy living. Consistency across coaches is important. Provide healthy snacks and drinks at all your events. Trips/camps are good for putting healthy habits into action. Use other athletes as examples.
Planning and Keeping Records	Coaches need to make a plan of what they are going to do and see how it works. Set daily, weekly goals that will motivate me. Take small steps at a time—progress slowly. Have a routine of daily activities. Adapt the exercise activities to suit my needs. Write down my training and gym workouts. Give the athlete time to improve their health.	Identify an athlete’s weak points. Take measures, such as weight, time to run 60 meters. Draw up an individual action plan for an athlete. Keep it simple and avoid it becoming a test or chore. Provide advice/handouts/videos on what to do. Have them keep a daily diary of activities—food, exercise. Use fitness tracker App on Smart-phone/Fit Bit. Families can double check their activities and records.
Help from others	There are health workers who can help. The gym is open for everyone—when we go it makes it more inclusive and accessible. Having a personal trainer is helpful. People at my gym are very friendly.	Refer to doctor, health clinics or hospital. Contact with local gyms. Identify other athletes living nearby. Join other community sporting activities. Find a neighbor, relative willing to buddy the athlete.

## Data Availability

The datasets presented in this article are not readily available because of assurances of confidentiality given to participants. Permissions would have to obtained from the participants for their information to be shared and applicants would be expected to cover the costs involved in doing so. Requests to access the datasets should be directed to the corresponding author.

## Abbreviations

The following abbreviations are used in this manuscript:

LMIC	Low- and Middle-Income Countries
NC	National Coordinator
PC	Project Coordinator
SO	Special Olympics
SOI	Special Olympics International
UN	United Nations
WHO	World Health Organization
